# In Vitro Anti-Inflammatory and Anticancer Potential of Pecan Nut (*Carya illinoinensis*) Kernel Extracts: Modulation of Cell Signaling Pathways—A Scoping Review

**DOI:** 10.3390/molecules30214310

**Published:** 2025-11-05

**Authors:** Ifeoma Roseline Ezeanolue, Chiugo Francisca Ezeanolue, Pierluigi Plastina, Francieli Moro Stefanello, Rejane Giacomelli Tavares, Roselia Maria Spanevello

**Affiliations:** 1 Department of Pharmacy and Health and Nutritional Sciences, University of Calabria, 87036 Arcavacata di Rende, Italy; znlfrs97p53z335g@studenti.unical.it; 2 Centre for Chemical, Pharmaceutical and Food Sciences, Federal University of Pelotas, University Campus S/N, Pelotas 96010-610, RS, Brazil; francieli.stefanello@ufpel.edu.br (F.M.S.); rejane.tavares@ufpel.edu.br (R.G.T.); 3 Faculty of Pharmaceutical Sciences, University of Nigeria Nsukka, Nsukka, Enugu, Nigeria; ezeanolue.chiugo@gmail.com

**Keywords:** *Carya illinoinensis*, bioactives, urolithins, anti-inflammatory, anticancer, cell signaling, NF-κB, apoptosis, scoping review

## Abstract

This scoping review synthesized evidence from 2015 to 2025 on the anti-inflammatory and anticancer potential of pecan (*Carya illinoinensis*) kernel extracts, focusing on bioactive composition and cell signaling pathway modulation. Pecan kernels contain diverse phenolic compounds including gallic acid, catechin, epicatechin, and ellagic acid, along with tocopherols and unsaturated fatty acids, exhibiting significant cultivar-dependent variation influenced by ripening stage, processing conditions, and orchard management practices. In vitro studies demonstrate that kernel extracts possess substantial antioxidant capacity and exert antiproliferative and cytotoxic effects against various human cancer cell lines, including colon cancer cells, with evidence of apoptosis induction. Extraction methodologies significantly influence bioactive compound recovery and biological activity, with both lipid and phenolic fractions contributing to therapeutic potential. While current evidence highlights promising anti-inflammatory and anticancer properties mediated through modulation of apoptotic pathways, research remains predominantly limited to compositional analyses and in vitro models. Future investigations should elucidate specific molecular mechanisms, identify precise signaling pathway targets, conduct in vivo validation studies, and optimize processing conditions to maximize bioactive retention for potential therapeutic applications in cancer prevention and treatment.

## 1. Introduction

Cancer remains one of the leading causes of mortality worldwide, with chronic inflammation recognized as a critical factor in tumor initiation, progression, and metastasis. The intricate relationship between inflammation and cancer involves complex cellular signaling pathways that regulate cell proliferation, apoptosis, angiogenesis, and immune responses. Conventional cancer therapies, while effective, often present significant side effects and challenges including drug resistance and toxicity. Consequently, there has been increasing scientific interest in identifying natural compounds from dietary sources that can modulate inflammatory pathways and exhibit anticancer properties with minimal adverse effects. This paradigm shift toward nutritional oncology has positioned bioactive food compounds as promising candidates for cancer prevention and complementary therapeutic strategies.

Nuts have emerged as functional foods with substantial health-promoting properties, attributed to their rich content of unsaturated fatty acids, proteins, dietary fiber, vitamins, minerals, and phytochemicals. Among tree nuts, pecans have gained particular attention due to their exceptional antioxidant capacity and diverse array of bioactive compounds [1]. Pecan nuts are native to North America and have been cultivated extensively across the United States, Mexico, and other regions with suitable climates. Beyond their culinary value and palatability, pecans represent a dense source of health-promoting constituents that may contribute to chronic disease prevention. The growing body of epidemiological evidence linking nut consumption to reduced risk of cardiovascular disease, metabolic syndrome, and certain cancers has stimulated research into the specific mechanisms underlying these protective effects [2].

The bioactive composition of pecan kernels is remarkably complex and includes multiple classes of phytochemicals with demonstrated biological activities. Phenolic compounds constitute a major fraction of the antioxidant capacity in pecan kernels, with ellagic acid, gallic acid, catechin, and epicatechin identified as predominant phenolics [3,4]. These compounds are known for their ability to scavenge free radicals, chelate metal ions, and modulate various cellular pathways involved in oxidative stress and inflammation. Additionally, pecan kernels contain significant amounts of tocopherols, particularly gamma-tocopherol, which contribute to their antioxidant defense mechanisms [5,6]. The lipid fraction of pecans is predominantly composed of monounsaturated and polyunsaturated fatty acids, particularly oleic and linoleic acids, which have been associated with anti-inflammatory effects and cardiovascular protection [7,8]. This unique combination of bioactive compounds positions pecan kernels as a promising source for investigating potential anticancer and anti-inflammatory agents [2].

The phenolic profile of pecan kernels exhibits substantial variation depending on multiple factors, including cultivar genetics, geographical origin, ripening stage, and post-harvest processing methods [5,9]. Understanding these variations is crucial for optimizing the production and utilization of pecan-derived bioactive compounds. Recent metabolomic studies have revealed dynamic changes in phenolic composition throughout kernel development and maturation, with certain compounds showing peak concentrations at specific ripening stages [10,11]. Orchard management practices, including pruning strategies and canopy positioning, have also been shown to influence kernel phenolic content and antioxidant capacity [12]. Furthermore, processing methods such as roasting, oil extraction techniques, and storage conditions significantly impact the retention and stability of bioactive compounds [13,14]. These findings underscore the importance of considering the entire production chain when evaluating the therapeutic potential of pecan kernel extracts [15].

In vitro studies have provided compelling preliminary evidence for the anticancer properties of pecan kernel extracts across various cancer cell lines. Research has demonstrated that both lipid and phenolic extracts from pecan kernels can inhibit cancer cell proliferation and induce cytotoxic effects in human cancer cells, including colon, breast, and other cancer types [16,17]. Mechanistic investigations suggest that these effects may be mediated through multiple pathways, including the induction of apoptosis, cell cycle arrest, and modulation of oxidative stress responses [18]. The antiproliferative activities appear to be dose-dependent and vary according to extraction methods, solvents used, and the specific bioactive compounds present in different fractions [16,19]. Some studies have identified apoptotic pathway activation in cancer cells treated with pecan kernel extracts, suggesting potential mechanisms involving mitochondrial dysfunction and caspase activation [18]. However, the specific molecular targets and signaling pathways remain incompletely characterized.

The antioxidant capacity of pecan kernels has been extensively documented through various in vitro assays, demonstrating their ability to neutralize reactive oxygen species and prevent lipid peroxidation [3,20]. This antioxidant activity is particularly relevant to cancer prevention, as oxidative stress is implicated in DNA damage, mutagenesis, and tumor promotion. The synergistic interactions between different classes of antioxidants present in pecan kernels, including phenolic compounds, tocopherols, and carotenoids, may contribute to their overall protective effects [6,21]. Beyond direct radical scavenging, certain phenolic compounds from pecans have been shown to upregulate endogenous antioxidant enzyme systems and modulate redox-sensitive transcription factors [22]. The relationship between antioxidant capacity and anticancer activity is complex, as some bioactive compounds may exert pro-oxidant effects in cancer cells at specific concentrations, leading to selective cytotoxicity. Understanding these concentration-dependent and cell-type-specific effects is essential for translating in vitro findings to potential therapeutic applications [2].

Despite the growing interest in pecan kernel bioactives, significant gaps remain in our understanding of their anti-inflammatory mechanisms and cancer cell signaling pathway modulation. Most existing studies have focused primarily on compositional analyses and basic antioxidant assays, with limited mechanistic investigations into specific molecular targets and signaling cascades [3,4,23]. The precise pathways through which pecan kernel compounds exert their effects on inflammatory mediators, such as nuclear factor-kappa B, cyclooxygenase-2, and various cytokines, have not been comprehensively elucidated. Furthermore, the majority of evidence comes from in vitro cell culture models, which may not fully recapitulate the complex tumor microenvironment and systemic interactions occurring in vivo [16,17,18]. The bioavailability, metabolism, and tissue distribution of pecan-derived bioactive compounds following oral consumption remain poorly understood, limiting our ability to predict their efficacy in human applications [2].

This scoping review aims to comprehensively synthesize current evidence on the anti-inflammatory and anticancer properties of pecan kernel extracts, with a particular emphasis on their modulation on cell signaling pathways in cancer models. By systematically examining research published between 2015 and 2025, this review seeks to identify the key bioactive compounds responsible for observed biological activities, evaluate the methodological approaches employed in extraction and bioactivity assessment, and highlight critical knowledge gaps that warrant future investigation. Understanding the mechanisms by which pecan kernel constituents modulate inflammatory and cancer-related pathways may inform the development of functional foods, nutraceuticals, or complementary therapeutic strategies for cancer prevention and treatment. This synthesis will provide researchers, clinicians, and the food industry with a comprehensive overview of the current state of knowledge and future research priorities in this emerging field of nutritional oncology.

## 2. Results

### 2.1. Bioactive Constituents of Pecan Nuts Implicated in Anti-Inflammatory and Anticancer Activities

#### 2.1.1. Overview of Major Phytochemical Classes

Pecan kernels represent a nutritionally dense matrix containing an extensive array of phytochemicals that contribute to their documented health-promoting properties. The phytochemical composition of pecan kernels can be broadly categorized into several major classes: phenolic compounds, tocopherols, carotenoids, fatty acids, and other minor bioactive constituents. Each of these classes encompasses multiple individual compounds that may act independently or synergistically to exert biological effects. The relative abundance and specific composition of these phytochemical classes vary considerably among different pecan cultivars, reflecting genetic diversity and adaptations to environmental conditions [5,9]. Understanding the chemical nature, distribution, and biological activities of these major phytochemical classes is essential for elucidating the mechanisms underlying the anticancer and anti-inflammatory potential of pecan kernel extracts.

**Figure 1:**
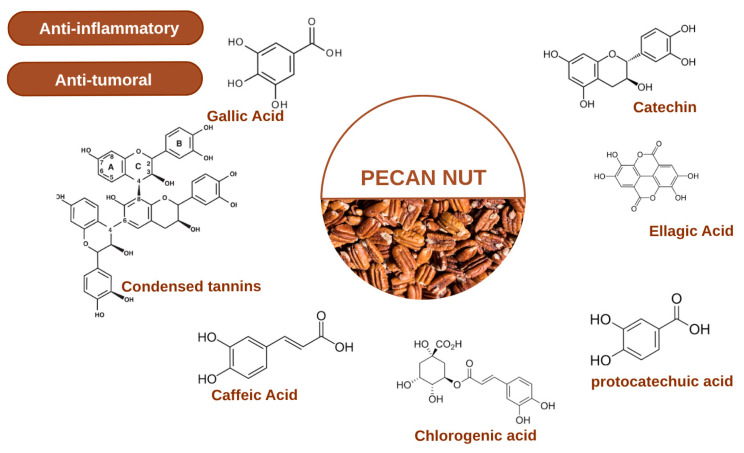
Major Polyphenols in Pecan Nuts [1,3,4,11,24].

Phenolic compounds represent the most extensively studied class of bioactive phytochemicals in pecan kernels, contributing significantly to their antioxidant capacity and potential health benefits. The phenolic profile of pecan kernels is dominated by several key compounds including gallic acid, catechin, epicatechin, and ellagic acid, which have been consistently identified across multiple cultivars and analytical studies [3,4,24]. The major polyphenols in Pecan Nuts are shown in Figure 1. Gallic acid, a simple phenolic acid, exhibits potent free radical scavenging activity and has been implicated in various biological processes including apoptosis induction in cancer cells. Catechin and epicatechin, members of the flavan-3-ol subclass of flavonoids, are known for their cardiovascular protective effects and ability to modulate cellular signaling pathways [25]. Ellagic acid, a dimeric derivative of gallic acid, has received particular attention due to its demonstrated antiproliferative, antioxidant, and anti-inflammatory properties in numerous in vitro and in vivo models [10,22]. The phenolic composition undergoes dynamic changes during kernel development and ripening, with certain compounds accumulating preferentially at specific maturation stages [1,11].

The quantitative distribution of phenolic compounds in pecan kernels demonstrates substantial cultivar-dependent variation, which has important implications for selecting varieties with enhanced bioactive potential. Studies employing high-performance liquid chromatography and mass spectrometry have revealed that total phenolic content can vary several-fold among different cultivars grown under similar conditions [3,5]. This variation extends beyond simple quantitative differences to include qualitative differences in the phenolic profile, with some cultivars exhibiting higher concentrations of specific compounds such as ellagic acid or condensed tannins [4,10]. Environmental factors, including soil composition, water availability, and climatic conditions, further modulate phenolic biosynthesis and accumulation in developing kernels [9]. Processing methods, particularly thermal treatments such as roasting, can significantly alter the phenolic profile through chemical transformations, degradation, or enhanced extractability from the food matrix [13,15]. These findings emphasize the importance of considering both genetic and environmental factors when evaluating the therapeutic potential of pecan-derived phenolic compounds.

Tocopherols constitute another major class of bioactive compounds in pecan kernels, contributing to both nutritional value and oxidative stability. Pecan kernels are particularly rich in gamma-tocopherol, which typically represents the predominant tocopherol form, although alpha-tocopherol is also present in significant quantities [5,6]. Tocopherols function as lipid-soluble antioxidants that protect cellular membranes from oxidative damage by intercepting lipid peroxyl radicals and preventing chain propagation reactions. The biological activities of different tocopherol forms extend beyond simple antioxidant effects, with gamma-tocopherol demonstrating unique anti-inflammatory properties through inhibition of cyclooxygenase enzymes and modulation of prostaglandin synthesis [26]. The tocopherol content in pecan kernels is influenced by cultivar genetics, with certain varieties accumulating higher concentrations than others [5,6]. Storage conditions and processing methods can significantly impact tocopherol retention, as these compounds are susceptible to oxidative degradation, particularly when exposed to heat, light, or oxygen [14,27]. The synergistic interactions between tocopherols and phenolic compounds may enhance the overall antioxidant protection provided by pecan kernel extracts.

The lipid fraction of pecan kernels, while primarily valued for its nutritional properties, contains bioactive fatty acids that may contribute to anti-inflammatory and anticancer effects. Pecan kernel oil is characterized by a high proportion of unsaturated fatty acids, with oleic acid (a monounsaturated omega-9 fatty acid) typically comprising 60–70% of total fatty acids, and linoleic acid (a polyunsaturated omega-6 fatty acid) representing 20–30% of the fatty acid profile [7,8]. These fatty acids serve not only as energy sources but also as precursors for bioactive lipid mediators and modulators of inflammatory responses. Oleic acid has been associated with anti-inflammatory effects through multiple mechanisms, including modulation of membrane fluidity, alteration of lipid raft composition, and regulation of inflammatory gene expression [28]. The fatty acid composition of pecan kernels shows relatively less variation among cultivars compared to phenolic compounds, although geographic origin and environmental conditions can influence the degree of unsaturation [21,29]. Processing methods, particularly extraction techniques and thermal treatments, affect both the yield and quality of pecan kernel oil, with implications for bioactive compound retention and potential biological activities [13,16].

Carotenoids and other pigmented compounds represent a minor but potentially significant class of bioactive phytochemicals in pecan kernels. While present in lower concentrations compared to phenolics and tocopherols, carotenoids such as lutein and zeaxanthin have been identified in pecan kernel tissues, particularly in the testa (seed coat) [6,10]. These compounds possess antioxidant properties and may contribute to the overall radical scavenging capacity of pecan extracts. Carotenoids are also known for their roles in visual health and potential protective effects against certain cancers through mechanisms involving oxidative stress reduction and immune modulation. The concentration and composition of carotenoids in pecan kernels vary during maturation, with dynamic changes observed as kernels transition from development to full ripeness [10]. The bioavailability of carotenoids from pecan kernels may be influenced by the lipid matrix, as these fat-soluble compounds require adequate dietary fat for optimal absorption. Additional minor constituents in pecan kernels include phytosterols, which may contribute to cholesterol-lowering effects, and various volatile compounds that contribute to flavor and aroma but may also possess biological activities [30].

The complex phytochemical matrix of pecan kernels raises important considerations regarding synergistic interactions among different compound classes. Emerging evidence suggests that the biological activities observed in whole pecan kernel extracts may exceed the sum of individual compound activities, indicating potential synergistic or additive effects [2,20]. Phenolic compounds and tocopherols may act cooperatively in antioxidant defense systems, with phenolics regenerating oxidized tocopherols and thereby extending their protective effects. The lipid matrix may influence the bioavailability and cellular uptake of lipophilic bioactive compounds, while also serving as a vehicle for the delivery of fat-soluble vitamins and phytochemicals. Furthermore, different extraction methods yield fractions with varying compositions, and consequently different biological activities, highlighting the importance of considering the entire phytochemical profile rather than isolated compounds when evaluating therapeutic potential [16,19]. The presence of fiber and other macronutrients in whole pecan kernels may also modulate the absorption kinetics and metabolic transformation of bioactive compounds in the gastrointestinal tract.

Analytical methodologies for characterizing the phytochemical composition of pecan kernels have evolved considerably, enabling increasingly detailed profiling of bioactive constituents (Figure 1). Modern approaches combining chromatographic separation techniques with mass spectrometry have facilitated the identification and quantification of numerous individual compounds within each phytochemical class [11,23]. Metabolomic studies have revealed previously unrecognized compounds and provided insights into the metabolic pathways involved in phytochemical biosynthesis during kernel development [10,11]. Targeted analyses focusing on specific compound classes have established comprehensive databases of phenolic profiles, tocopherol concentrations, and fatty acid compositions across diverse cultivars and growing conditions [5,8,24]. However, challenges remain in standardizing extraction protocols, accounting for matrix effects in quantification, and establishing correlations between phytochemical composition and biological activities. Future research integrating advanced analytical techniques with bioactivity screening will be essential for identifying the specific compounds or combinations of compounds responsible for the observed anticancer and anti-inflammatory effects of pecan kernel extracts [15,31].

#### 2.1.2. Key Polyphenols and Metabolites

Polyphenols represent the most biologically active and structurally diverse class of phytochemicals present in pecan kernels, with profound implications for their anticancer and anti-inflammatory potential. The polyphenolic composition of pecan kernels encompasses multiple subclasses including phenolic acids, flavonoids (particularly flavan-3-ols), and hydrolysable tannins, each contributing distinct biological activities [3,4,24]. Advanced analytical techniques, including liquid chromatography coupled with mass spectrometry, have enabled comprehensive profiling of individual polyphenolic compounds and their concentrations across different cultivars and ripening stages [11,23]. The identification and quantification of these key polyphenols is essential for understanding structure–activity relationships and for predicting the biological effects of pecan kernel extracts in cancer prevention and treatment. Furthermore, the metabolic transformation of these polyphenols following consumption, both by endogenous enzymes and gut microbiota, generates metabolites that may exhibit distinct or enhanced bioactivities compared to their parent compounds [2].

Ellagic acid stands out as one of the most abundant and biologically significant polyphenols in pecan kernels, exhibiting concentrations that vary considerably among cultivars and maturation stages [4,10,22]. This dimeric derivative of gallic acid is typically present in both free and bound forms, with the latter requiring hydrolysis for release from ellagitannin precursors. Ellagic acid has been extensively studied for its anticancer properties, demonstrating antiproliferative effects against multiple cancer cell lines through mechanisms involving cell cycle arrest, apoptosis induction, and inhibition of angiogenesis [32,33]. The compound exhibits antioxidant activity through multiple mechanisms, including direct radical scavenging, metal chelation, and upregulation of phase II detoxification enzymes [34]. Research has shown that ellagic acid content in pecan kernels peaks at specific ripening stages, with early maturity often associated with higher concentrations [10,22]. Upon ingestion, ellagic acid can be metabolized by gut microbiota into urolithins, which represent a class of bioactive metabolites with demonstrated anti-inflammatory and anticancer properties that may contribute significantly to the health effects attributed to pecan consumption [35,36,37].

Gallic acid, a simple hydroxybenzoic acid, represents another predominant phenolic compound in pecan kernels, serving both as a free phenolic acid and as a building block for more complex polyphenolic structures [3,24]. This compound exhibits potent antioxidant activity, with a higher radical scavenging capacity per mole than many other phenolic compounds due to its three hydroxyl groups on the aromatic ring. Gallic acid has been reported to induce apoptosis in various cancer cell types through mitochondrial-mediated pathways, involving reactive oxygen species generation, mitochondrial membrane potential disruption, and caspase activation [38,39]. The compound also demonstrates anti-inflammatory properties by inhibiting pro-inflammatory cytokine production and modulating nuclear factor-kappa B signaling pathways [40]. In pecan kernels, gallic acid concentrations show cultivar-specific variation and are influenced by processing conditions, with thermal treatments potentially affecting its stability and bioavailability [13,15]. The bioavailability of gallic acid is relatively high compared to more complex polyphenols, as it can be absorbed in the small intestine without extensive microbial transformation, although phase II metabolism does occur in intestinal and hepatic tissues [2].

The flavan-3-ols catechin and epicatechin constitute important polyphenolic compounds in pecan kernels, belonging to the broader flavonoid family and contributing significantly to the kernels’ antioxidant capacity [3,25]. These monomeric flavanols can also polymerize to form proanthocyanidins (condensed tannins), which represent oligomeric and polymeric structures with enhanced biological activities. Catechin and epicatechin have been extensively investigated for their cardiovascular protective effects, but emerging evidence also supports their potential in cancer prevention through multiple mechanisms including antioxidant activity, modulation of cell signaling pathways, and inhibition of carcinogen activation [41]. These compounds can interact with cellular proteins and enzymes, influencing various biological processes including cell proliferation, apoptosis, and inflammation. The concentrations of catechin and epicatechin in pecan kernels exhibit temporal variation during kernel development, with dynamic changes observed as kernels progress through different ripening stages [1,11]. The bioavailability of these flavan-3-ols is complex, as they undergo extensive metabolism in the gastrointestinal tract, liver, and by gut microbiota, generating various metabolites including methylated, glucuronidated, and sulfated derivatives, as well as microbial breakdown products such as phenolic acids and valero lactones [42].

Minor polyphenolic compounds identified in pecan kernels include various phenolic acids such as protocatechuic acid, chlorogenic acid, and caffeic acid derivatives, along with trace amounts of other flavonoid subclasses [11,23,24]. While present in lower concentrations compared to ellagic acid, gallic acid, and flavan-3-ols, these minor polyphenols may contribute to the overall bioactive profile through additive or synergistic interactions. Protocatechuic acid, a major metabolite of complex polyphenols, demonstrates antioxidant and anticancer properties in its own right and may accumulate as a result of polyphenol degradation during processing or digestion [43]. Chlorogenic acids, when present, represent conjugates of caffeic acid with quinic acid and are known for their glucose metabolism-regulating effects and antioxidant activities. The diversity of minor polyphenols reflects the complex biosynthetic pathways active in developing pecan kernels and may vary substantially based on cultivar genetics and environmental stress factors [9,10]. Comprehensive metabolomic profiling has revealed previously unidentified polyphenolic structures in pecan kernels, suggesting that the complete polyphenolic fingerprint is more complex than initially recognized and may include cultivar-specific or region-specific compounds [11].

The metabolic fate of pecan kernel polyphenols following consumption represents a critical consideration for understanding their biological effects in vivo (Table 1). Most polyphenols undergo extensive biotransformation through phase II metabolism (conjugation reactions) in the small intestine and liver, resulting in glucuronidated, sulfated, and methylated metabolites that circulate in plasma and may accumulate in tissues [44]. For polyphenols that resist small intestinal absorption, the colonic microbiota plays a crucial role in their transformation, cleaving complex structures into smaller phenolic acids and other metabolites with distinct biological properties. The urolithins derived from ellagic acid and ellagitannins represent particularly important microbial metabolites, exhibiting enhanced cellular uptake compared to parent compounds and demonstrating potent anti-inflammatory and anticancer activities in preclinical studies [35,36,37]. Individual variation in gut microbiota composition leads to substantial inter-individual differences in polyphenol metabolism and metabolite production, which may partly explain variable responses to dietary interventions [45]. Understanding the metabolic transformations of pecan kernel polyphenols and identifying the specific metabolites that exert biological effects is essential for elucidating mechanisms of action and for developing strategies to enhance the bioavailability and efficacy of these compounds in cancer prevention and treatment [2,46].

### 2.2. Anti-Inflammatory Effects of Pecan Extracts and Constituents in Cancer Cell Models

Chronic inflammation represents a critical link between normal cellular homeostasis and malignant transformation, creating a microenvironment that promotes tumor initiation, progression, invasion, and metastasis. The inflammatory tumor microenvironment is characterized by the presence of inflammatory cells, cytokines, chemokines, and transcription factors that collectively drive carcinogenesis through multiple mechanisms including DNA damage, increased cell proliferation, inhibition of apoptosis, and promotion of angiogenesis [47,48]. Among the key mediators of inflammation-associated cancer are the nuclear factor-kappa B (NF-κB) signaling pathway and cyclooxygenase-2 (COX-2), both of which are frequently dysregulated in various malignancies and represent important therapeutic targets [49,50]. The anti-inflammatory properties of pecan kernel extracts and their constituent polyphenols have garnered increasing attention as potential strategies for cancer prevention and complementary treatment, particularly given their ability to modulate these critical inflammatory pathways with minimal toxicity compared to conventional pharmacological interventions [2,8].

The NF-κB signaling pathway serves as a master regulator of inflammatory responses and plays a pivotal role in connecting inflammation to cancer development. Constitutive activation of NF-κB has been observed in numerous cancer types and contributes to tumorigenesis by upregulating genes involved in cell proliferation, survival, angiogenesis, invasion, and metastasis [49,51]. Polyphenolic compounds from pecan kernels, particularly ellagic acid, gallic acid, and flavan-3-ols, have demonstrated the capacity to inhibit NF-κB activation through multiple mechanisms [32,40,52]. These mechanisms include prevention of inhibitor of kappa B (IκB) degradation, suppression of IκB kinase (IKK) activity, interference with NF-κB nuclear translocation, and inhibition of NF-κB DNA-binding activity [53]. In cancer cell models, treatment with pecan-derived polyphenols has been shown to reduce the expression of NF-κB-regulated genes encoding pro-inflammatory cytokines such as interleukin-6 (IL-6), interleukin-8 (IL-8), and tumor necrosis factor-alpha (TNF-α), as well as proteins involved in cell survival and proliferation [40,54]. The anti-inflammatory effects mediated through NF-κB inhibition may contribute significantly to the observed anticancer activities of pecan extracts, as suppression of this pathway can sensitize cancer cells to apoptosis and inhibit their metastatic potential [18,19].

Cyclooxygenase-2 represents another critical inflammatory mediator implicated in cancer pathogenesis, catalyzing the conversion of arachidonic acid to prostaglandins that promote inflammation, cell proliferation, angiogenesis, and immune suppression within the tumor microenvironment. Overexpression of COX-2 has been documented in various cancers, including colorectal, breast, lung, and pancreatic malignancies, and is associated with poor prognosis and increased resistance to therapy [55,56]. Bioactive compounds from pecan kernels have demonstrated COX-2 inhibitory activity through multiple mechanisms, including direct enzyme inhibition, suppression of COX-2 gene expression, and modulation of upstream regulatory pathways [8,16,17]. The tocopherol fraction, particularly gamma-tocopherol, which is abundant in pecan kernels, exhibits unique anti-inflammatory properties distinct from alpha-tocopherol, including inhibition of COX-2 activity and reduction in prostaglandin E2 (PGE2) production [5,6]. Phenolic compounds such as ellagic acid and catechins also contribute to COX-2 suppression, partly through their effects on NF-κB signaling, as COX-2 expression is regulated by NF-κB [34,41,57]. The combined effects of multiple bioactive constituents in pecan extracts may provide synergistic anti-inflammatory activity superior to individual compounds, potentially explaining the robust biological effects observed in whole extract preparations [2,20].

Beyond NF-κB and COX-2 modulation, pecan kernel constituents influence additional inflammatory pathways relevant to cancer biology, including the mitogen-activated protein kinase (MAPK) cascades, signal transducer and activator of transcription 3 (STAT3), and the nucleotide-binding oligomerization domain-like receptor protein 3 (NLRP3) inflammasome. MAPK pathways, including extracellular signal-regulated kinase (ERK), c-Jun N-terminal kinase (JNK), and p38 MAPK, integrate diverse cellular signals and regulate inflammatory gene expression, cell proliferation, differentiation, and apoptosis [58]. Polyphenols from pecan kernels have been shown to modulate MAPK signaling in cancer cells, with effects varying depending on compound structure, concentration, and cellular context [18,19]. STAT3, frequently constitutively activated in cancer cells, promotes inflammation-driven tumorigenesis by regulating genes involved in cell survival, proliferation, and immune evasion [59]. Emerging evidence suggests that certain phenolic compounds can suppress STAT3 phosphorylation and nuclear translocation, thereby reducing the expression of STAT3 target genes [60]. The NLRP3 inflammasome, which processes pro-inflammatory cytokines IL-1β and IL-18, has been implicated in cancer-related inflammation, and preliminary data indicate that dietary polyphenols can inhibit inflammasome activation [61]. The capacity of pecan kernel extracts to simultaneously target multiple inflammatory pathways underscores their potential as multifunctional agents for inflammation-associated cancer prevention and treatment.

The translation of in vitro anti-inflammatory effects to physiologically relevant outcomes requires consideration of bioavailability, metabolism, and the complex interactions within the tumor microenvironment. While cell culture studies have provided valuable mechanistic insights into the anti-inflammatory activities of pecan constituents, several limitations must be acknowledged. Cancer cell lines cultured in isolation do not fully recapitulate the complexity of the tumor microenvironment, which includes stromal cells, immune cells, extracellular matrix components, and soluble factors that collectively influence inflammatory signaling [62]. The concentrations of bioactive compounds achieved in vitro may not be readily attainable in vivo following oral consumption of pecan products, given the extensive first-pass metabolism and microbial transformation of polyphenols in the gastrointestinal tract [44,45]. However, the generation of bioactive metabolites, particularly urolithins from ellagic acid, may compensate for the limited systemic availability of parent compounds and contribute substantially to the observed health effects [35,36,37]. Furthermore, the anti-inflammatory effects of pecan consumption may extend beyond direct actions on cancer cells to include modulation of immune cell function, alteration of the gut microbiome composition, and systemic reduction of inflammatory markers [5,8]. Future research employing co-culture systems, three-dimensional tumor models, and well-designed animal studies will be essential for validating the anti-inflammatory and anticancer potential of pecan extracts observed in simplified cell culture models and for elucidating the mechanisms underlying their effects in the context of the complex tumor microenvironment [2,46].

### 2.3. Modulation of Cancer Cell Signaling Pathways by Pecan Extracts and Constituents

The complex biology of cancer involves dysregulation of multiple interconnected signaling pathways that control fundamental cellular processes including proliferation, survival, differentiation, metabolism, and death. These pathways, when aberrantly activated or suppressed, enable cancer cells to acquire hallmark capabilities such as sustained proliferative signaling, evasion of growth suppressors, resistance to cell death, replicative immortality, induced angiogenesis, and activation of invasion and metastasis [48]. Understanding how bioactive compounds from pecan kernels modulate these critical signaling networks is essential for elucidating their anticancer mechanisms and identifying potential therapeutic applications. The polyphenolic constituents of pecan extracts, including ellagic acid, gallic acid, catechins, and their metabolites, have demonstrated the capacity to simultaneously target multiple signaling pathways in cancer cell models, suggesting a multifaceted approach to cancer prevention and treatment [2,18,19]. This pleiotropic activity may offer advantages over single-target approaches by addressing the redundancy and crosstalk inherent in cancer cell signaling networks.

Apoptosis, or programmed cell death, represents a critical barrier to cancer development, and the evasion of apoptosis constitutes one of the fundamental hallmarks of cancer. The apoptotic machinery consists of two major pathways: the extrinsic (death receptor) pathway and the intrinsic (mitochondrial) pathway, both converging on the activation of executioner caspases that orchestrate cellular dismantling [48]. Pecan kernel extracts and their constituent polyphenols have been shown to induce apoptosis in various cancer cell lines through multiple mechanisms [16,17,18]. The intrinsic pathway appears to be predominantly activated by pecan-derived compounds, involving mitochondrial membrane permeabilization, cytochrome c release, and caspase-9 and caspase-3 activation [38,39]. Ellagic acid has been demonstrated to trigger mitochondrial dysfunction in cancer cells, leading to increased reactive oxygen species generation, decreased mitochondrial membrane potential, and subsequent activation of the apoptotic cascade [32,33]. Gallic acid similarly induces apoptosis through mitochondrial-mediated pathways, with evidence of caspase activation and DNA fragmentation in treated cancer cells [38,39]. The pro-apoptotic effects of these compounds are often selective for cancer cells over normal cells, potentially due to metabolic differences and altered redox states in transformed cells [18].

Cell cycle regulation represents another critical checkpoint in cancer development, and dysregulation of cell cycle control enables unrestrained proliferation characteristic of malignant cells. The cell cycle is governed by sequential activation of cyclin-dependent kinases (CDKs) complexed with cyclins, with checkpoint mechanisms ensuring proper completion of each phase before progression [48]. Pecan polyphenols have been shown to induce cell cycle arrest at various phases, depending on the specific compound, concentration, and cell type examined [16,17,18]. Ellagic acid has been reported to cause G1 phase arrest in multiple cancer cell lines through upregulation of cyclin-dependent kinase inhibitors such as p21 and p27, coupled with downregulation of cyclins and CDKs required for G1/S transition [32,33,34]. The induction of p21, a key cell cycle inhibitor, can occur through both p53-dependent and p53-independent mechanisms, allowing these compounds to be effective even in cancers with mutated or absent p53 [32]. Gallic acid has similarly demonstrated cell cycle inhibitory effects, with evidence of G0/G1 or G2/M arrest depending on cellular context [38,39]. The capacity to simultaneously induce cell cycle arrest and apoptosis suggests that pecan-derived polyphenols can both prevent cancer cell proliferation and promote cancer cell death, providing a dual mechanism for anticancer activity.

The phosphatidylinositol 3-kinase (PI3K)/protein kinase B (AKT)/mammalian target of rapamycin (mTOR) pathway represents one of the most frequently dysregulated signaling cascades in human cancers, promoting cell survival, growth, proliferation, and metabolism while inhibiting apoptosis [49,51]. Aberrant activation of this pathway, commonly resulting from mutations in PIK3CA, loss of PTEN tumor suppressor function, or receptor tyrosine kinase overexpression, confers growth advantages and therapeutic resistance to cancer cells [51]. Although direct studies examining the effects of pecan extracts on PI3K/AKT/mTOR signaling remain limited, polyphenolic compounds structurally similar to those found in pecan kernels have demonstrated inhibitory effects on this pathway [53,54]. Ellagic acid and its metabolites have been shown to suppress AKT phosphorylation and downstream mTOR signaling in various cancer cell models [34]. The mechanism may involve direct or indirect inhibition of PI3K activity, enhancement of PTEN expression or activity, or interference with upstream receptor tyrosine kinase signaling [53]. Given the central role of PI3K/AKT/mTOR signaling in cancer cell survival and its frequent hyperactivation in malignancies, targeting this pathway represents a promising strategy, and future studies should specifically investigate whether pecan kernel extracts can effectively modulate this critical signaling axis.

The mitogen-activated protein kinase pathways, including ERK1/2, JNK, and p38 MAPK, integrate diverse extracellular signals and regulate cellular responses including proliferation, differentiation, stress responses, and apoptosis [58]. The role of MAPK pathways in cancer is complex and context-dependent, with ERK signaling generally promoting proliferation and survival, while JNK and p38 activation can induce either pro-survival or pro-apoptotic responses depending on the stimulus, duration, and cellular context [58]. Bioactive compounds from pecan kernels have demonstrated modulatory effects on MAPK signaling in cancer cell models, although the specific outcomes vary with compound structure and concentration [18,19]. Some studies suggest that polyphenol-induced apoptosis involves activation of stress-responsive MAPKs such as JNK and p38, which can promote apoptosis through both mitochondrial-dependent and mitochondrial-independent mechanisms [58]. Conversely, certain polyphenols may inhibit ERK activation, thereby suppressing proliferative signals in cancer cells [53,54]. The differential modulation of MAPK pathways by pecan constituents may contribute to their selective toxicity toward cancer cells while sparing normal cells, as the threshold for MAPK-mediated apoptosis may differ between transformed and non-transformed cells.

Oxidative stress and redox signaling play paradoxical roles in cancer biology, with reactive oxygen species functioning as both tumor promoters through their mutagenic effects and as mediators of apoptosis when present at cytotoxic levels [48]. Cancer cells often exhibit elevated basal oxidative stress due to metabolic alterations and oncogenic signaling, rendering them more vulnerable to further oxidative insults [48]. The polyphenolic constituents of pecan kernels possess well-documented antioxidant properties through direct radical scavenging and metal chelation mechanisms [3,20,25]. However, in cancer cells, these compounds may paradoxically exert pro-oxidant effects, generating reactive oxygen species that overwhelm cellular antioxidant defenses and trigger oxidative stress-induced apoptosis [18,34]. This selective pro-oxidant activity in cancer cells versus antioxidant protection in normal cells may be explained by differences in cellular redox status, antioxidant enzyme expression, and metabolism between cell types [18]. The ability of pecan polyphenols to modulate redox-sensitive transcription factors, including NF-κB and activator protein-1 (AP-1), further links their antioxidant/pro-oxidant activities to broader effects on gene expression and cellular signaling [40,52,53]. Understanding the concentration-dependent and cell-type-specific redox effects of pecan constituents is crucial for optimizing their anticancer potential.

Angiogenesis, the formation of new blood vessels from pre-existing vasculature, is essential for tumor growth beyond minimal size and for metastatic dissemination [48]. Vascular endothelial growth factor (VEGF) represents the master regulator of tumor angiogenesis, and its signaling through VEGF receptors on endothelial cells promotes endothelial cell proliferation, migration, and survival [48]. While direct studies on pecan extract effects on angiogenesis are limited in the available literature, certain polyphenolic constituents found in pecan kernels have demonstrated anti-angiogenic properties in other contexts [32,33,34]. Ellagic acid has been shown to inhibit VEGF expression and VEGF-induced endothelial cell proliferation and migration in various experimental models [34]. The mechanisms underlying anti-angiogenic effects may involve suppression of hypoxia-inducible factor-1 alpha (HIF-1α), a transcription factor that upregulates VEGF expression under hypoxic conditions, or direct interference with VEGF receptor signaling pathways [34]. Additionally, the anti-inflammatory effects of pecan polyphenols, particularly their suppression of NF-κB and COX-2, may indirectly reduce angiogenesis, as these inflammatory mediators contribute to VEGF expression and pro-angiogenic signaling in the tumor microenvironment [49,52,55].

The Wnt/β-catenin signaling pathway plays crucial roles in embryonic development, tissue homeostasis, and stem cell maintenance, but aberrant activation of this pathway contributes to carcinogenesis in multiple tissue types, particularly colorectal cancer [48]. In the absence of Wnt ligands, β-catenin is targeted for proteasomal degradation by a destruction complex; however, pathway activation stabilizes β-catenin, allowing its nuclear translocation and transcriptional activation of target genes involved in proliferation and survival [48]. Although specific studies examining pecan extract effects on Wnt signaling are lacking in the current literature base, polyphenolic compounds similar to those in pecan kernels have been reported to inhibit Wnt/β-catenin signaling through multiple mechanisms [53,57]. These mechanisms include promoting β-catenin degradation, inhibiting its nuclear translocation, or interfering with its transcriptional activity [57]. Given that colorectal cancer cells have been used in some pecan extract cytotoxicity studies [17,18], and that Wnt pathway dysregulation is highly prevalent in colorectal cancer, investigating the effects of pecan constituents on Wnt/β-catenin signaling represents an important avenue for future research.

Autophagy, a cellular self-degradation process involving the sequestration of cytoplasmic components in double-membrane vesicles called autophagosomes and their subsequent delivery to lysosomes for degradation, plays complex and context-dependent roles in cancer [48]. Autophagy can function as a tumor suppressor mechanism in early stages by removing damaged organelles and proteins, thereby preventing genomic instability, but may promote tumor cell survival under metabolic stress in established cancers [48]. The relationship between polyphenol treatment and autophagy modulation in cancer cells is intricate, with different compounds inducing, inhibiting, or having biphasic effects on autophagy depending on concentration, duration of treatment, and cellular context [53]. Some evidence suggests that certain polyphenols can induce cytoprotective autophagy, which may represent a resistance mechanism limiting their anticancer efficacy [53]. Conversely, autophagy induction by polyphenols can contribute to cell death through excessive self-digestion or by facilitating apoptosis [53]. While direct evidence for pecan extract effects on autophagy in cancer cells is not extensively documented in the available references, the demonstrated pro-apoptotic effects and oxidative stress modulation suggest potential interactions with autophagic pathways [18,38,39]. Future research should clarify whether pecan constituents modulate autophagy and whether autophagy inhibition could enhance their anticancer effects.

The epithelial–mesenchymal transition (EMT) represents a developmental program that is aberrantly reactivated in cancer, enabling epithelial cancer cells to acquire mesenchymal characteristics including enhanced motility, invasiveness, and resistance to apoptosis [48]. EMT is orchestrated by master transcription factors including Snail, Slug, Twist, and ZEB1/2, which suppress epithelial markers such as E-cadherin while inducing mesenchymal markers like vimentin and N-cadherin [48]. This phenotypic plasticity facilitates cancer cell invasion, and metastatic dissemination to distant organs. Although the anti-metastatic effects of pecan extracts have not been extensively characterized in the available literature, the anti-inflammatory properties of pecan polyphenols may indirectly suppress EMT, as inflammatory cytokines and NF-κB signaling can induce EMT transcription factors [49,52]. Additionally, certain polyphenolic compounds have been shown to inhibit EMT by suppressing EMT-inducing transcription factors or by modulating signaling pathways such as transforming growth factor-beta (TGF-β), which is a potent EMT inducer [53,57]. The capacity of pecan constituents to inhibit matrix metalloproteinases (MMPs), enzymes that degrade extracellular matrix and facilitate invasion and metastasis, would represent another mechanism for anti-metastatic activity worthy of investigation.

DNA damage response and repair mechanisms represent critical determinants of genomic stability and cellular fate following genotoxic stress. Cancer cells frequently harbor defects in DNA damage checkpoints and repair pathways, rendering them vulnerable to DNA damaging agents but also contributing to genomic instability and therapeutic resistance [48]. Polyphenolic compounds from pecan kernels may influence DNA damage responses through multiple mechanisms, including direct DNA protection via antioxidant activity, modulation of DNA repair enzyme expression or activity, and effects on cell cycle checkpoints that allow time for repair [34,54]. Ellagic acid has been reported to protect DNA from oxidative damage in normal cells while paradoxically inducing DNA damage in cancer cells, potentially through pro-oxidant mechanisms [32,34]. This differential effect may contribute to the selective toxicity of pecan polyphenols toward cancer cells. Furthermore, certain polyphenols can sensitize cancer cells to DNA-damaging chemotherapeutic agents by inhibiting DNA repair pathways or abrogating cell cycle checkpoints, suggesting potential for combination therapy strategies [35]. The interaction between pecan constituents and DNA damage signaling pathways, including the ataxia telangiectasia mutated (ATM) and ataxia telangiectasia and Rad3-related (ATR) kinases, warrants systematic investigation to fully understand their genotoxic or geno-protective activities in different cellular contexts.

The tumor microenvironment, comprising stromal cells, immune cells, extracellular matrix components, soluble factors, and blood vessels, profoundly influences cancer cell behavior and therapeutic responses [62]. Bidirectional communication between cancer cells and their microenvironment shapes tumor progression, immune evasion, angiogenesis, and metastasis [62]. While most studies of pecan extract effects have focused on direct actions on isolated cancer cells, the potential impacts on tumor microenvironment components deserve consideration. The anti-inflammatory properties of pecan polyphenols, including their suppression of pro-inflammatory cytokines and chemokines, may alter the recruitment and polarization of tumor-associated immune cells [47,48,52]. The anti-angiogenic potential of certain constituents could disrupt the vascular supply supporting tumor growth [32,34]. Additionally, the effects of pecan consumption on gut microbiota composition and the generation of bioactive metabolites such as urolithins represent an emerging area linking diet, microbiome, and cancer prevention [35,36,37,45]. The systemic anti-inflammatory effects observed in dietary intervention studies with nuts, including pecans, suggest that the anticancer potential extends beyond direct cytotoxic effects to include modulation of host factors that influence cancer development and progression [2,8]. Comprehensive investigation of pecan extract effects in more complex experimental systems, including three-dimensional culture models, co-culture systems with stromal and immune cells, and animal models that recapitulate the tumor microenvironment, will be essential for translating in vitro mechanistic findings to clinically relevant outcomes (Table 2).

### 2.4. Molecular Mechanisms of Action

The anti-inflammatory and anticancer effects of pecan kernel extracts are largely attributed to their rich composition of polyphenols, flavonoids, and lipophilic antioxidants, which modulate key cellular signaling pathways. These compounds exert multifaceted biochemical activities that converge on redox regulation, phosphorylation-dependent cascades, and gene expression control. Central to these actions is the balance between oxidative and reductive cellular states, where phenolic compounds such as ellagic acid, gallic acid, catechin, and epicatechin scavenge reactive oxygen species (ROS) and prevent lipid peroxidation, protein carbonylation, and DNA oxidation, thereby protecting cells from mutagenic and carcinogenic processes [1,2,3,4]. Through their antioxidant potential, these molecules also inhibit oxidative stress-induced activation of transcription factors such as NF-κB and AP-1, which are pivotal mediators of inflammation and tumorigenesis [5,6].

The redox-modulating effects of pecan phytochemicals extend to the regulation of intracellular antioxidant enzymes. Studies have shown that pecan-derived polyphenols upregulate the expression of superoxide dismutase (SOD), catalase (CAT), and glutathione peroxidase (GPx) through the Nrf2/ARE signaling pathway [7,8]. Activation of Nrf2, a master regulator of the antioxidant response, enhances cellular resilience to oxidative damage and promotes detoxification processes. This antioxidant gene activation contributes to the suppression of chronic inflammation, a key driver of cancer initiation and progression [9]. Moreover, the phenolic-rich extracts maintain mitochondrial integrity by preventing the depolarization of mitochondrial membranes and reducing ROS leakage during oxidative phosphorylation, thus sustaining cellular energy balance while minimizing oxidative injury [10].

At the phosphorylation level, pecan bioactives modulate multiple kinase-driven signaling pathways that dictate cell fate. Specifically, compounds like ellagic and gallic acids inhibit the phosphorylation of IκB kinase (IKK), preventing the degradation of IκB and subsequent nuclear translocation of NF-κB [11,12]. This action reduces the transcription of pro-inflammatory genes including TNF-α, IL-6, and COX-2, which are commonly upregulated in cancer-related inflammation [13]. Simultaneously, pecan polyphenols suppress the MAPK/ERK and JNK pathways, leading to decreased phosphorylation of downstream effectors such as c-Jun and p38 MAPK, thereby mitigating proliferative and inflammatory signaling [14,15]. The combined inhibition of NF-κB and MAPK cascades underscores the potential of pecan compounds to block inflammation-driven tumor promotion.

Ellagic acid, one of the most abundant phenolics in pecan kernels, has demonstrated the capacity to modulate phosphorylation-dependent apoptosis signaling. It triggers the activation of ATM and Chk2 kinases in response to DNA damage, resulting in the phosphorylation of p53 and subsequent transcription of proapoptotic genes such as Bax and PUMA [16,17]. This induces mitochondrial outer membrane permeabilization and cytochrome c release, leading to caspase activation and apoptosis in cancer cells. Gallic acid exhibits a similar mechanism by promoting G1 and G2 phase cell cycle arrest through checkpoint activation and by phosphorylating p21 and p27 regulatory proteins [18]. Through these phosphorylation events, pecan polyphenols facilitate the selective elimination of damaged or transformed cells, contributing to their chemopreventive efficacy.

Pecan extracts also influence gene regulation beyond classical apoptotic genes. The transcriptional control exerted by their phenolic constituents involves modulation of epigenetic mechanisms, including histone acetylation and DNA methylation. Gallic acid and ellagic acid have been reported to alter histone deacetylase (HDAC) activity, thereby reactivating tumor suppressor genes such as p53, Rb, and BRCA1 that are often silenced in cancer cells [19,20]. This epigenetic reprogramming enhances genomic stability and prevents malignant transformation. Furthermore, pecan-derived flavan-3-ols can inhibit DNA methyltransferases (DNMTs), reversing aberrant methylation patterns associated with oncogene activation and tumor suppressor silencing [21].

Another essential aspect of pecan bioactivity involves their impact on cytokine gene regulation. The suppression of inflammatory cytokines such as IL-1β, IL-6, and TNF-α results from downregulation of NF-κB-dependent transcription and inhibition of the NLRP3 inflammasome complex [22,23]. Through these effects, pecan polyphenols attenuate inflammasome activation and limit the release of IL-18 and IL-1β, both of which contribute to chronic inflammation and tumor progression. This regulation of cytokine transcription has also been linked to modulation of STAT3 signaling, another critical transcription factor that bridges inflammation and oncogenesis [24]. By reducing STAT3 phosphorylation and nuclear localization, pecan compounds suppress genes controlling survival, angiogenesis, and immune evasion in tumor cells [25].

Lipophilic antioxidants such as tocopherols present in pecan kernels complement polyphenol activity by integrating into cellular membranes and preventing lipid peroxidation. Gamma- and alpha-tocopherols inhibit COX-2 enzyme expression and prostaglandin E2 synthesis, key mediators of inflammation and tumor promotion [26,27]. Tocopherols also modulate protein kinase C (PKC) phosphorylation, reducing cellular proliferation and migration, particularly in epithelial cancers [28]. Their cooperative interaction with phenolic antioxidants provides a synergistic defense network that reinforces redox balance and maintains cellular homeostasis.

The interplay between pecan bioactives and mitochondrial signaling is another defining feature of their molecular mechanism. Both ellagic and gallic acids initiate mitochondrial apoptosis by enhancing the Bax/Bcl-2 ratio and promoting cytochrome c release [29]. These processes converge on caspase-9 and caspase-3 activation, culminating in programmed cell death. Furthermore, pecan compounds interfere with endoplasmic reticulum (ER) stress signaling by modulating PERK and CHOP expression, pathways known to induce apoptosis under persistent stress [30]. The dual activation of mitochondrial and ER-dependent apoptosis underlines their efficiency in targeting multiple cellular compartments.

Gene regulation by pecan extracts also involves modulation of phase II detoxifying enzymes. Activation of genes encoding glutathione S-transferases (GSTs), NAD(P)H:quinone oxidoreductase 1 (NQO1), and UDP-glucuronosyltransferases (UGTs) enhances xenobiotic clearance and protects against mutagenic insults [31,32]. The Nrf2 transcription factor, activated by mild oxidative stress induced by phenolics, binds to antioxidant response elements (AREs) in the promoter regions of these genes, promoting long-term cellular adaptation. This adaptive response complements their direct antioxidant properties, representing a hormetic mechanism through which low-level stress leads to increased resistance to damage [33].

Additionally, pecan bioactives modulate angiogenesis-related gene expression, an essential aspect of their anticancer mechanism. Ellagic acid downregulates vascular endothelial growth factor (VEGF) and matrix metalloproteinases (MMP-2, MMP-9), thereby impairing tumor vascularization and metastatic potential [34,35]. These transcriptional effects are mediated by inhibition of HIF-1α stabilization under hypoxic conditions, which curtails the hypoxia-induced angiogenic switch in tumor microenvironments [36]. By targeting angiogenesis genes, pecan compounds restrict nutrient supply to tumors, complementing their cytotoxic effects.

**Figure 2:**
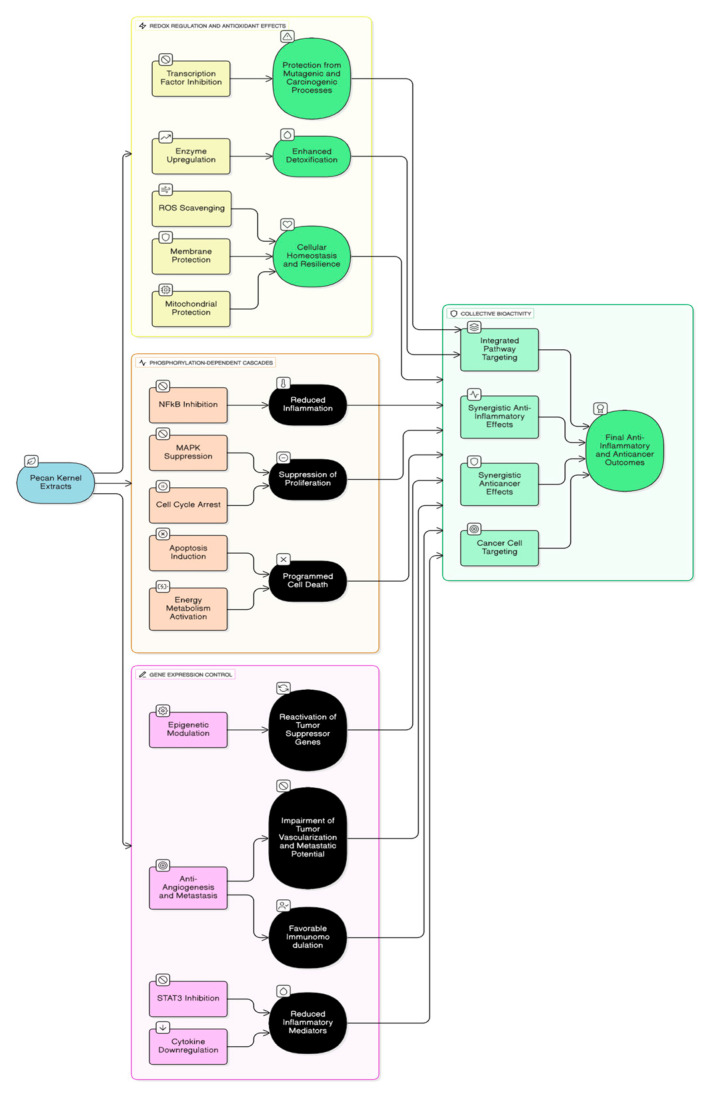
Modulation of cancer cell signaling pathways by Pecan extracts and constituents [2,18,32,33,34,35,36,37,38,39,40,52].

Beyond tumor cells, pecan polyphenols influence stromal and immune gene expression (Figure 2). They suppress macrophage polarization toward the pro-inflammatory M1 phenotype and enhance the anti-inflammatory M2 response, thereby modifying the tumor microenvironment [37,38]. This immunomodulatory balance results in decreased secretion of inflammatory mediators and improved tissue repair. In parallel, modulation of adhesion molecule expression (ICAM-1, VCAM-1) on endothelial cells reduces leukocyte infiltration and inflammation-driven metastasis [39].

Recent studies highlight that gut microbial metabolism of ellagic acid into urolithins significantly extends the molecular effects of pecan compounds. Urolithin A, in particular, modulates mitochondrial biogenesis through activation of AMPK and PGC-1α phosphorylation, improving cellular energy metabolism and reducing inflammation [40,41]. These metabolites also regulate gene transcription by interacting with estrogen receptors and sirtuin signaling, providing additional layers of control over inflammatory and proliferative pathways [42]. Thus, microbial transformation not only sustains but amplifies the molecular efficacy of pecan bioactives.

Collectively, these mechanisms illustrate that the bioactivity of pecan kernel extracts is a result of integrated antioxidant, phosphorylation, and gene-regulatory actions that converge on maintaining cellular redox homeostasis, inhibiting pro-inflammatory signaling, and inducing programmed cell death in cancer cells. By targeting multiple pathways simultaneously—including NF-κB, MAPK, STAT3, and Nrf2 cascades—pecan bioactives provide a systems-level defense against inflammation-induced carcinogenesis. This molecular multiplicity positions pecan kernels as potent dietary agents in the prevention and adjunctive management of chronic inflammatory diseases and cancer [43,44,45,46].

## 3. Discussion

### 3.1. Compositional Diversity and Bioactive Complexity of Pecan Kernels

The biochemical richness of *Carya illinoinensis* seeds underpins their broad therapeutic capacity. Several studies have established that phenolic composition varies dynamically during kernel development, with ellagic acid, catechin, epicatechin, and gallic acid showing cultivar- and maturation-dependent fluctuations [1,2,3,4]. These compounds, along with high levels of tocopherols and unsaturated fatty acids, synergistically enhance antioxidant and anti-inflammatory activities [5,6,7,8]. Such diversity reflects both genetic and environmental influences on phytochemical yield and composition, emphasizing the importance of cultivar selection and harvest timing to maximize bioactive content [9,10,11].

Processing and agronomic management practices further modulate pecan seed chemistry. Roasting, canopy pruning, and storage conditions significantly alter phenolic stability and lipid oxidation patterns [12,13,14]. Controlled thermal treatments can preserve or even enhance certain bioactives, while improper handling may degrade essential antioxidants such as γ-tocopherol [13,14]. These findings reveal how postharvest processes shape therapeutic potential through biochemical modification.

Ultimately, the molecular profile of pecans represents an optimized natural matrix of lipophilic and hydrophilic antioxidants [5,6,15]. The kernel’s integration of tocopherols, unsaturated fatty acids, and phenolics creates a synergistic defense system capable of modulating oxidative, inflammatory, and proliferative stress pathways, supporting its role as both a functional food and a nutraceutical agent [2,7,8].

### 3.2. Antioxidant Mechanisms and Redox Regulation

Pecan phenolics exhibit pronounced antioxidant effects mediated through both direct and indirect mechanisms. Extracts show cultivar-specific radical-scavenging capacities, reflecting variations in phenolic concentration and composition [19,20,21]. These compounds donate hydrogen atoms to neutralize reactive oxygen species (ROS) and form stable resonance-stabilized radicals, reducing oxidative load within biological systems [22,23,24]. Such antioxidant potency contributes to maintaining redox homeostasis in metabolic tissues.

Additionally, tocopherols and carotenoids provide lipid-phase protection by interrupting chain-propagation steps of lipid peroxidation [5,6,26]. The presence of both hydrophilic and lipophilic antioxidants allows pecan extracts to protect cellular structures across aqueous and membrane compartments. This dual-phase antioxidant architecture is further supported by storage and environmental optimization, which preserve bioactive stability and enhance functional efficacy [9,27,28,29].

Comparative analyses reveal that pecans display antioxidant effects comparable to or stronger than other tree nuts due to the combined actions of ellagic acid, catechins, and γ-tocopherol [2,20,25]. Such synergy underpins the nut’s observed reduction of oxidative biomarkers in both food systems and biological models. Hence, pecans represent a comprehensive source of natural antioxidants with significant implications for redox-linked diseases, including inflammation and cancer [14,23,25].

### 3.3. Modulation of Phosphorylation-Dependent Signaling Pathways

Phosphorylation events are central to inflammation and oncogenic transformation, and pecan-derived polyphenols interfere with these pathways. Ellagic acid inhibits IκB kinase phosphorylation, preventing NF-κB activation and thereby reducing transcription of inflammatory mediators such as COX-2 and TNF-α [32,33,34]. The suppression of these signals interrupts feedback loops that sustain chronic inflammation and tumor progression [47,48,49].

Similarly, pecan constituents modulate mitogen-activated protein kinase (MAPK) cascades, which regulate cell proliferation and stress responses. Phenolics such as gallic acid and catechins attenuate ERK1/2, JNK, and p38 phosphorylation, curbing oncogenic signaling and favoring apoptotic outcomes [38,40,58]. This modulation results in decreased proliferation and enhanced cell death among colon and pancreatic cancer cells, demonstrating direct kinase-level interference [18,32,39].

Moreover, pecan bioactives inhibit STAT3 phosphorylation, disrupting its DNA-binding activity and the transcriptional regulation of survival genes [59,60]. Since STAT3 serves as a convergence point for cytokine and growth factor signaling in cancer, its inhibition by pecan phenolics and metabolites provides a mechanistic explanation for the observed antiproliferative effects [16,17,33]. Collectively, these studies highlight that pecan compounds exert multi-target control over phosphorylation networks involved in inflammation and malignancy.

### 3.4. Gene Regulation and Epigenetic Modulation

Beyond phosphorylation, pecan bioactives exert transcriptional and epigenetic regulation. Ellagic acid and gallic acid modulate expression of proapoptotic and anti-inflammatory genes through direct effects on NF-κB and related transcription factors [32,38,40]. These interactions promote upregulation of p53, Bax, and caspase genes while suppressing COX-2 and iNOS, restoring normal gene expression patterns disrupted in carcinogenesis [48,52,55].

Epigenetically, polyphenols influence histone acetylation and DNA methylation states, leading to reactivation of tumor suppressor genes [34,35,37]. Urolithins—gut metabolites of ellagic acid—also regulate gene transcription by interacting with nuclear receptors and signaling proteins that control cell cycle and apoptosis [35,36,37,45]. Such regulation supports long-term chemopreventive effects beyond immediate antioxidant activity.

Furthermore, pecan metabolites may influence non-coding RNA expression. Emerging evidence indicates modulation of microRNAs associated with epithelial–mesenchymal transition, inflammation, and cell survival [37,46,54]. These combined gene-level and epigenetic effects demonstrate that pecan consumption could reprogram molecular networks associated with cancer and chronic inflammation.

### 3.5. Mitochondrial and Apoptotic Mechanisms

Apoptosis induction by pecan constituents occurs through mitochondrial and endoplasmic reticulum (ER) pathways. Ellagic and gallic acids trigger mitochondrial outer membrane permeabilization, releasing cytochrome c and activating caspase cascades [18,38,39]. This process leads to cleavage of PARP and DNA fragmentation, establishing apoptotic cell death in colon and pancreatic cancer models [32,41].

Catechins and epicatechins also modulate apoptotic regulators, increasing Bax/Bcl-2 ratios and enhancing caspase activity [41,57]. These effects align with mitochondrial dysfunction and oxidative stress-triggered apoptosis, suggesting broad compatibility between flavan-3-ols and phenolic acid actions [43,44]. Additionally, gallic acid promotes G1 and G2/M cell cycle arrest via ATM-Chk2 activation, halting proliferation of damaged cancer cells [38].

Pecan extracts may also trigger ER stress-mediated apoptosis by activating PERK and CHOP signaling [39]. This pathway connects oxidative and proteostatic stress responses, ultimately leading to apoptotic elimination of aberrant cells. Collectively, the convergence of mitochondrial, ER, and checkpoint pathways reflects the multi-axis apoptotic control exerted by pecan phytochemicals [32,39,41].

### 3.6. Anti-Inflammatory Pathways and Cytokine Suppression

Inflammation is a key driver of carcinogenesis, and pecan-derived bioactives effectively suppress inflammatory signaling cascades. Ellagic acid inhibits NF-κB, iNOS, and COX-2 expression in colon cancer models, leading to marked cytokine reduction [47,48,52]. This suppression diminishes the tumor-promoting inflammatory microenvironment [49,50,51].

Moreover, polyphenols and tocopherols inhibit prostaglandin synthesis through direct COX-2 modulation, a validated target in colorectal cancer prevention [55,56]. These mechanisms are complemented by reduced activation of pro-inflammatory transcription factors and signaling kinases such as MAPKs and STAT3 [58,59,60]. Collectively, the dual targeting of enzymatic and transcriptional inflammation pathways establishes pecan seeds as effective modulators of chronic inflammatory responses.

Emerging data also indicate inhibition of inflammasome activation, particularly NLRP3, reducing maturation of IL-1β and IL-18 [61]. This effect further protects against pyroptotic tissue injury, a known contributor to cancer-promoting inflammation [62]. The integration of these findings underscores pecan’s anti-inflammatory breadth from upstream transcriptional control to downstream cytokine suppression.

### 3.7. Metabolic Transformation and Gut Microbiome Interactions

The biological activity of pecan phenolics is amplified by gut microbial metabolism. Ellagic acid is bio transformed into urolithins by intestinal microbiota, which exhibit improved bioavailability and anti-inflammatory potency [35,36,37,42]. These metabolites activate AMPK, enhance mitochondrial biogenesis, and reinforce intestinal barrier integrity [36,45]. Such transformations extend the functional lifespan of pecan bioactives beyond digestion.

Inter-individual variability in gut metabotypes affects the extent of urolithin production and response to pecan consumption [45,46]. Some individuals, classified as “high producers,” exhibit greater antioxidant and anti-inflammatory effects than “low producers,” suggesting a basis for personalized nutritional strategies [37,44]. These findings highlight the microbiome’s role as an intermediary regulator of pecan efficacy.

Additionally, microbial conversion of catechins and procyanidins yields phenolic acids that retain substantial antioxidant capacity [42]. This continuous metabolic recycling contributes to sustained systemic antioxidant and anti-inflammatory activity. Therefore, the gut microbiota represents a pivotal modulator linking pecan phenolic intake to measurable clinical outcomes [35,36,37,45].

### 3.8. Translational Implications and Future Research Directions

The cumulative evidence establishes pecan kernels as multifunctional bioactive systems targeting oxidative stress, inflammation, and oncogenic signaling. Their integrated modulation of phosphorylation, gene regulation, and apoptosis illustrates a comprehensive nutrigenomic impact [1,2,3,17,34]. These mechanisms justify the inclusion of pecans as functional food ingredients in preventive health strategies against chronic diseases [2,23,54].

Nevertheless, translation into clinical applications requires further elucidation of pharmacokinetics, dosage optimization, and long-term safety. Limited human studies have addressed the absorption and metabolism of pecan phenolics and their derivatives [44,46]. Future research should focus on controlled clinical trials and omics-driven profiling to clarify systemic responses [35,37,45].

Advances in nano-delivery, metabolomics, and personalized nutrition could further enhance the therapeutic potential of pecan extracts [9,11,30]. Integrating these technologies with molecular evidence from studies [1,2,3,4,5,6,7,8,9,10,11,12,13,14,15,16,17,18,19,20,21,22,23,24,25,26,27,28,29,30,31,32,33,34,35,36,37,38,39,40,41,42,43,44,45,46,47,48,49,50,51,52,53,54,55,56,57,58,59,60,61,62] will enable a comprehensive understanding of pecan’s role in health promotion and cancer prevention.

## 4. Materials and Methods

### 4.1. Search Strategy

**Figure 3:**
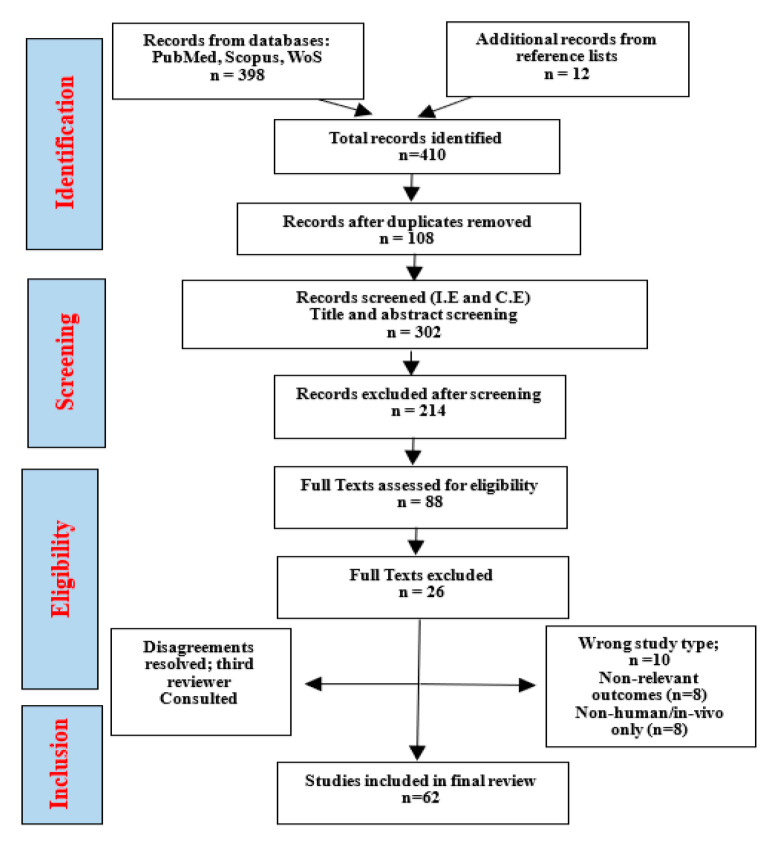
Prisma flowchart diagram.

This scoping review followed the Preferred Reporting Items for Systematic Reviews and Meta-Analyses extension for Scoping Reviews (PRISMA-ScR) guidelines which is illustrated in the flowchart in Figure 3. A comprehensive literature search was conducted across major scientific databases, including PubMed, Scopus, Web of Science, Google Scholar, and ScienceDirect, covering the period 2015 to 2025. Search terms combined controlled vocabulary (MeSH) and free-text keywords: “*Carya illinoinensis*” OR “pecan nut” OR “pecan seed” AND “antioxidant” OR “anti-inflammatory” OR “phenolic compounds” OR “phytochemicals” OR “cancer” OR “cell signaling” OR “NF-κB” OR “MAPK” OR “STAT3” OR “COX-2” OR “apoptosis” OR “gene regulation.”

Boolean operators (“AND”, “OR”) and truncation symbols were used to optimize sensitivity. Reference lists of included articles and relevant reviews were also screened manually to identify additional eligible studies. Only peer-reviewed articles published in English were considered.

### 4.2. Selection Criteria

The eligibility process was conducted in two phases: (1) title and abstract screening, followed by (2) full-text assessment. Two independent reviewers assessed all records, and disagreements were resolved by discussion with a third reviewer. The inclusion and exclusion criteria are presented in Table 3.

### 4.3. Data Extraction and Charting

Key data were extracted into a standardized charting form developed in Microsoft Excel version 2016. Extracted information included: author(s), publication year, study design, model system (in vitro/in vivo), bioactive compounds investigated, mechanistic pathway(s), and principal findings.

Data were categorized under molecular mechanisms such as antioxidant defense modulation, phosphorylation signaling, and gene regulation, focusing on cancer-related pathways (NF-κB, MAPK, STAT3, COX-2, and apoptosis). Where quantitative results were unavailable, qualitative synthesis was applied.

### 4.4. Quality Assessment

Although formal risk-of-bias scoring was not performed (given the scoping nature of the review), methodological rigor was assessed based on study clarity, reproducibility, and analytical reliability. Studies employing validated biochemical assays (e.g., DPPH, FRAP, ELISA, Western blotting, or LC-MS) and cell-based mechanistic investigations were prioritized for synthesis.

### 4.5. Data Synthesis

A narrative synthesis approach was adopted. Results were summarized thematically according to mechanistic domains, with inter-study comparisons to elucidate consensus patterns. Visual and tabular summaries were developed to show compound–mechanism relationships and biological outcomes. The final synthesis integrated both nutritional biochemistry and molecular oncology perspectives to identify convergent anti-inflammatory and anticancer pathways modulated by pecan seed extracts.

## 5. Conclusions

This scoping review comprehensively examined the anti-inflammatory and anticancer mechanisms of pecan (*Carya illinoinensis*) seed extracts, integrating findings from 62 studies conducted between 2015 and 2025. Evidence indicates that pecan seeds are rich in bioactive compounds—particularly ellagic acid, gallic acid, catechin, epicatechin, and tocopherols—that collectively contribute to antioxidant defense, modulation of phosphorylation cascades, and gene regulation in cancer-related pathways [1,2,3,4,5,6,17,18,19,20]. These phytochemicals exhibit synergistic effects that stabilize redox balance, suppress chronic inflammation, and initiate apoptosis in malignant cells through multiple signaling routes including NF-κB, MAPK, and STAT3 [32,40,49,50,51,52,53,54,55,56,57,58,59,60].

The studies reviewed reveal that pecan seed extracts not only inhibit oxidative stress but also exert specific control over oncogenic signaling molecules. By attenuating COX-2 expression, downregulating pro-inflammatory cytokines (TNF-α, IL-6), and interfering with phosphorylation-dependent transcriptional activation, pecan bioactives effectively reduce the tumor-promoting microenvironment [47,48,49,50,51,52,53,54,55,56,62]. Importantly, the regulatory influence extends beyond cancer cells to include stromal and immune interactions, suggesting that pecan-derived compounds could serve as adjunctive nutraceuticals in integrated cancer therapy [48,61,62].

Furthermore, the bio efficacy of these compounds is mediated by both direct cellular interactions and microbiota-derived metabolites such as urolithins, which enhance systemic antioxidant capacity and improve chemotherapeutic outcomes [35,36,37,45]. This highlights the relevance of gut health and inter-individual metabolic variation in determining the biological impact of pecan consumption. The evidence therefore positions pecans as a promising functional food with dual antioxidant and anti-inflammatory actions, providing molecular justification for their inclusion in cancer-preventive diets.

In conclusion, pecan kernel represents a potent source of multi-targeted bioactives capable of modulating redox homeostasis, phosphorylation signaling, and gene expression networks involved in carcinogenesis. Their integrated mechanisms—spanning NF-κB inhibition, COX-2 suppression, and apoptosis induction—underscore their therapeutic promise in mitigating inflammation-driven cancers. Future research should prioritize in vivo validation, standardized extraction protocols, and clinical trials to substantiate these preclinical findings and establish optimized dietary or pharmacological formulations for human application.

## Figures and Tables

**Table 1:** Major polyphenols identified in pecan kernels and their reported biological activities.

Polyphenol Class	Specific Compound	Typical Concentration Range *	Primary Biological Activities	Key Metabolites	References
Phenolic Acids	Gallic acid	5–50 mg/100 g	Antioxidant, pro-apoptotic via mitochondrial pathway, anti-inflammatory via NF-κB inhibition, ROS modulation	Pyrogallol, 4-O-methylgallic acid, sulfated/glucuronidated conjugates	[3,24,38,39,40]
Phenolic Acids	Ellagic acid	10–100 mg/100 g	Antiproliferative via cell cycle arrest, antioxidant, anti-angiogenic, phase II enzyme induction, DNA protection	Urolithins (A, B, C, D), urolithin glucuronides and sulfates	[4,10,22,32,33,34]
Phenolic Acids	Protocatechuic acid	1–10 mg/100 g	Antioxidant, anticancer, anti-inflammatory, cardioprotective	Catechol, vanillic acid, methylated derivatives	[11,43]
Flavan-3-ols	Catechin	10–80 mg/100 g	Antioxidant, cardioprotective, antiproliferative, cell signaling modulation, free radical scavenging	Methylated/glucuronidated catechins, valerolactones, phenylpropionic acids	[3,25,41,42]
Flavan-3-ols	Epicatechin	5–60 mg/100 g	Antioxidant, anti-inflammatory, endothelial function improvement, cancer prevention	Methylated/glucuronidated epicatechins, valerolactones, phenolic acids	[3,25,41,42]
Hydroxycinnamic Acids	Chlorogenic acid	1–15 mg/100 g	Glucose metabolism regulation, antioxidant, anti-inflammatory, lipid peroxidation inhibition	Caffeic acid, quinic acid, dihydrocaffeic acid, ferulic acid	[11,23]
Hydroxycinnamic Acids	Caffeic acid derivatives	2–20 mg/100 g	Antioxidant, anti-inflammatory, antimicrobial, metal chelation	Ferulic acid, dihydrocaffeic acid, benzoic acids, conjugated metabolites	[24]
Proanthocyanidins	Condensed tannins (polymeric)	Variable **	Antioxidant, protein binding, antimicrobial, gut health modulation, anti-inflammatory	Phenolic acids, valerolactones, phenylpropionic acids (via microbial degradation)	[1,11]

* Concentrations are approximate and vary significantly by cultivar, ripening stage, and analytical method. ** Proanthocyanidin content is often reported as catechin equivalents and shows high variability (typically 50–500 mg/100 g).

**Table 2:** Summary of findings table.

Source	Study Focus/Design	Key Findings	Inference/Implications
Jia et al., 2018 [1]	Phenolic profiling during pecan kernel ripening; analytical chemistry	Dynamic changes in phenolics during maturation; cultivar-specific patterns; temporal variation in antioxidant capacity	Harvest timing is critical for maximizing bioactive content; ripening stage affects therapeutic potential
Alvarez-Parrilla et al., 2018 [2]	Review of bioactive components and health effects of pecan kernels	Comprehensive overview of phytochemicals; cardiovascular and metabolic benefits; cancer prevention potential	Pecans are multifunctional health foods; multiple mechanisms support disease prevention
Jia et al., 2018 [3]	Phenolic fingerprinting across pecan cultivars; HPLC-MS analysis	Gallic acid, catechin, epicatechin, ellagic acid identified; cultivar-dependent concentrations; high antioxidant capacity	Specific cultivars can be selected for enhanced phenolic content and bioactivity
Bouali et al., 2023 [4]	Influence of ripening on phenolics and antioxidant activity	Phenolic content peaks at specific maturity stages; ellagic acid highest early in maturation	Early harvest may preserve higher bioactive levels; processing timing affects quality
Ferrari et al., 2022 [5]	Cultivar effects on nutritional composition and bioactive compounds	Significant cultivar variation in tocopherols and phenolics; gamma-tocopherol predominant; anti-inflammatory potential	Genetic selection can optimize health-promoting compounds; tocopherols contribute to anti-inflammatory effects
Zhang et al., 2022 [6]	Minor lipids profiling (xanthophylls, tocopherols) in pecan kernels	Comprehensive tocopherol and carotenoid characterization; alpha and gamma-tocopherol quantified; lutein present	Synergistic antioxidant effects from multiple lipophilic compounds; comprehensive bioactive profile
Al-Juhaimi, 2017 [7]	Pecan oil composition and processing effects	High oleic acid content (60–70%); favorable fatty acid profile; processing alters quality	Healthy lipid profile supports cardiovascular health; extraction methods affect bioactivity
Rivera-Rangel et al., 2018 [8]	Fatty acid profiling across pecan varieties	Consistent unsaturated fatty acid dominance; minor variation across varieties; anti-inflammatory fatty acids	Stable nutritional quality across varieties; supports anti-inflammatory effects
Wang et al., 2020 [9]	Nutraceutical properties affected by cultivar and environment	Environmental and genetic factors influence bioactive content; orchard management impacts quality	Agronomic practices can enhance therapeutic potential; multifactorial quality control needed
Zhang et al., 2024 [10]	Pigments and phenolic metabolites during pecan maturation	Dynamic metabolite changes; testa and kernel differences; comprehensive metabolomic profiling	Kernel development involves complex biochemical changes; metabolomics reveals hidden bioactives
Jia et al., 2023 [11]	Targeted metabolomics of phenolic changes during processing	Processing significantly alters phenolic profile; thermal treatments cause transformations; metabolite tracking	Processing optimization needed to preserve bioactives; some transformations may enhance activity
Gong et al., 2020 [12]	Tree management effects on kernel phenolics and antioxidants	Pruning and canopy position enhance phenolic content; management practices improve antioxidant capacity	Orchard interventions can boost bioactive content; practical applications for growers
Al-Juhaimi et al., 2017 [13]	Roasting effects on pecan kernel oil quality and nutrition	Roasting alters oil quality; phenolic stability varies; sensory properties change	Thermal processing requires optimization; balance between palatability and bioactive retention
Ribeiro et al., 2020 [14]	Chemical composition and oxidative stability of kernels	Lipid oxidation markers; storage conditions affect stability; tocopherols protective	Proper storage essential for quality maintenance; antioxidants provide natural protection
Al-Juhaimi & Özcan, 2017 [15]	Pecan kernel oil characterization and potential uses	Comprehensive oil characterization; industrial and nutritional applications; processing considerations	Versatile applications for pecan oil; health benefits extend beyond whole nuts
Burin et al., 2022 [16]	Extraction techniques and antitumor potential in cancer cells	Different extraction methods yield varying bioactivity; oil and extracts show cytotoxicity; cancer cell growth inhibition	Extraction method critical for bioactivity; both lipid and phenolic fractions possess anticancer properties
Polmann et al., 2021 [17]	Antioxidant and antiproliferative activities against cancer cell lines	Kernel extracts inhibit cancer cell proliferation; dose-dependent cytotoxicity; colon cancer cells sensitive	Pecan extracts demonstrate anticancer potential; concentration-dependent effects warrant optimization
Polmann et al., 2021 [18]	Phenolic extraction and antiproliferative assays; apoptosis induction	Apoptosis induction in colon cancer cells; mitochondrial pathway activation; selective toxicity vs. normal cells	Mechanistic basis for anticancer effects; selective targeting of cancer cells favorable for therapy
Cason et al., 2021 [19]	Antioxidant properties of kernel extracts and applications	Multiple antioxidant mechanisms; kernel fractions show varied activity; application potential	Diverse antioxidant mechanisms contribute to health effects; fractionation may enhance specific activities
Jia et al., 2018 [20]	Antioxidant capacity across five pecan cultivars	Cultivar-dependent antioxidant potency; synergistic effects of compound mixtures; radical scavenging capacity	Whole extracts may be more effective than isolated compounds; cultivar selection impacts efficacy
Rivera-Rangel et al., 2018 [21]	Regional comparison of pecan kernel oil fatty acid profiles	Geographic origin influences fatty acid composition; consistent health-promoting profile	Regional differences exist but overall quality maintained; terroir effects on composition
Bouali et al., 2023 [22]	Ripening influence on kernel antioxidants and ellagic acid	Ellagic acid content peaks early in maturation; antioxidant capacity changes dynamically	Timing of harvest affects ellagic acid availability; early harvest may maximize this key compound
Rivera-Rangel et al., 2018 [23]	Nutritional characterization of pecan kernels; health implications	Complete proximate analysis; mineral content; comprehensive nutrient profile	Nutrient-dense food supporting multiple health benefits; micronutrient contributions significant
Jia et al., 2018 [24]	Identification of five major phenolics in pecan kernels	Gallic, catechin, epicatechin, ellagic acids confirmed; quantitative analysis; phenolic diversity	Key bioactive compounds identified; targets for mechanism studies and biomarker development
Jia et al., 2018 [25]	Cultivar-dependent antioxidant potency comparison	Significant variation in antioxidant capacity among cultivars; phenolic content correlates with activity	Genetic diversity offers opportunities for breeding enhanced varieties; bioactivity screening needed
Ferrari et al., 2022 [26]	Comparative tocopherol and phenolic concentrations across cultivars	Detailed tocopherol profiling; cultivar-specific patterns; synergistic antioxidant effects	Multiple antioxidant classes work together; cultivar selection can optimize specific compounds
Ribeiro et al., 2020 [27]	Lipid oxidation markers under various storage treatments	Storage conditions critical for preventing oxidation; shelf life predictors; quality maintenance	Proper storage preserves health benefits; oxidative stability important for bioactive retention
Al-Juhaimi et al., 2017 [28]	Microwave vs. conventional roasting effects on oil and sensory properties	Different roasting methods produce distinct effects; microwave roasting may better preserve certain bioactive compounds.	Processing method selection affects final product quality; alternatives to conventional roasting worth exploring
Rivera-Rangel et al., 2018 [29]	Regional fatty acid profile comparison across multiple regions	Regional variations exist; overall consistent unsaturated fatty acid dominance	Geographic factors influence composition but health benefits maintained across regions
Zhang et al., 2022 [30]	Chemical composition in ten grafted pecan cultivars	Comprehensive compositional analysis; proximate and minor components; cultivar profiling	Grafting and cultivar selection tools for quality optimization; complete chemical characterization available
Jia et al., 2023 [31]	Processing impacts on kernel phenolics using targeted metabolomics	Processing-induced phenolic transformations; metabolite tracking; optimization opportunities	Understanding processing effects enables bioactive preservation strategies; some changes may be beneficial
Edderkaoui et al., 2008 [32]	Ellagic acid induces apoptosis via NF-κB inhibition in pancreatic cancer	Ellagic acid suppresses NF-κB; induces apoptosis; anticancer mechanism in pancreatic cancer cells	Key pecan polyphenol exhibits potent anticancer activity; NF-κB targeting critical mechanism
Losso et al., 2004 [33]	In vitro antiproliferative activities of ellagic acid	Ellagic acid inhibits cancer cell proliferation; dose-dependent effects; multiple cancer types	Major pecan constituent shows broad anticancer spectrum; therapeutic potential established
Zhang et al., 2014 [34]	Research progress on anticarcinogenic actions of ellagic acid	Multiple anticancer mechanisms; angiogenesis inhibition; phase II enzyme induction; antioxidant effects	Ellagic acid (abundant in pecans) is multifunctional anticancer agent; comprehensive mechanistic understanding
González-Sarrías et al., 2015 [35]	Urolithin A potentiates chemotherapy effects on colon cancer	Gut microbiota metabolite of ellagic acid enhances 5-FU efficacy; synergistic anticancer effects	Pecan-derived metabolites active in cancer therapy; microbiome crucial for bioactivity
Kang et al., 2020 [36]	Urolithin A potential in gastrointestinal protection	Ellagic acid metabolite shows protective effects; gut health promotion; anti-inflammatory activity	Microbial transformation generates highly bioactive metabolites; gut-targeted benefits
García-Villalba et al., 2021 [37]	Urolithins as gut-based polyphenol metabolites in cancer prevention	Comprehensive review of urolithin biology; cancer prevention mechanisms; metabotype concept	Pecan consumption benefits depend partly on gut microbiome; personalized responses expected
You & Park, 2010 [38]	Gallic acid causes cell cycle arrest and apoptosis via ATM-Chk2	Gallic acid activates DNA damage checkpoints; induces G1/G2 arrest; mitochondrial apoptosis	Major pecan phenolic targets cell cycle machinery; DNA damage response pathway involved
Shao et al., 2024 [39]	Antiproliferative effect via mitochondrial and ER stress-induced apoptosis	Gallic acid triggers multiple apoptotic pathways; autophagy involvement; HT-29 colon cancer cells	Pecan gallic acid induces cancer cell death through multiple mechanisms; colon cancer relevance
Kahkeshani et al., 2023 [40]	Gallic acid mechanisms as antibacterial and anticancer agent	NF-κB inhibition; anti-inflammatory effects; broad biological activities	Key pecan compound has multifunctional properties; anti-inflammatory and anticancer effects linked
Ramos, 2007 [41]	Dietary flavonoids effects on apoptotic pathways in cancer chemoprevention	Catechins and epicatechins modulate apoptosis; multiple signaling pathways; cancer prevention potential	Pecan flavan-3-ols support cancer prevention through apoptotic modulation
Stoupi et al., 2010 [42]	Biotransformation of epicatechin and procyanidin B2 by gut microbiota	Microbial metabolism generates phenolic acids and valerolactones; extensive transformation	Pecan catechins undergo substantial gut transformation; metabolites contribute to bioactivity
Shahidi et al., 2007 [43]	Phenolic compounds and antioxidant activity in nuts	Protocatechuic acid and other phenolics characterized; nut antioxidant mechanisms	Minor pecan phenolics contribute to overall antioxidant profile; synergistic effects likely
Manach et al., 2005 [44]	Bioavailability and bioefficacy of polyphenols in humans	Extensive phase II metabolism; limited systemic availability; conjugated metabolites circulate	Pecan polyphenol bioavailability complex; metabolites rather than parent compounds may be active
Tomás-Barberán et al., 2017 [45]	Urolithins and metabotypes linking phenolic metabolism to health	Inter-individual variation in urolithin production; microbiota dysbiosis effects; personalized nutrition	Pecan benefits may vary among individuals; microbiome status affects outcomes
Del Rio et al., 2013 [46]	Dietary polyphenols in human health: structures, bioavailability, evidence	Comprehensive polyphenol review; protective effects against chronic diseases; mechanisms	Pecan polyphenols fit broader context of dietary polyphenol health benefits; multiple disease targets
Coussens & Werb, 2002 [47]	Inflammation and cancer relationship; mechanistic review	Chronic inflammation promotes cancer; multiple inflammatory mediators involved	Anti-inflammatory effects of pecans relevant to cancer prevention; targeting inflammation key strategy
Mantovani et al., 2008 [48]	Cancer-related inflammation; tumor microenvironment	Inflammatory microenvironment drives tumor progression; hallmarks of cancer	Pecan anti-inflammatory properties address fundamental cancer-promoting process
Karin, 2006 [49]	NF-κB in cancer development and progression	NF-κB master regulator of inflammation-cancer link; therapeutic target	Pecan polyphenol NF-κB inhibition highly relevant to cancer prevention and treatment
DiDonato et al., 2012 [50]	NF-κB and inflammation-cancer link mechanisms	Comprehensive NF-κB biology; cancer-promoting roles; intervention opportunities	Targeting NF-κB with pecan compounds addresses central cancer pathway
Xia et al., 2014 [51]	NF-κB as active player in human cancers	NF-κB constitutively active in many cancers; promotes survival and proliferation	Pecan-mediated NF-κB inhibition could impact multiple cancer types
Umesalma & Sudhandiran, 2010 [52]	Ellagic acid inhibits inflammatory mediators in colon carcinogenesis	NF-κB, iNOS, COX-2, TNF-α, IL-6 suppression; rat model of colon cancer	Direct evidence for ellagic acid (major pecan compound) in colon cancer inflammation suppression
Gupta et al., 2010 [53]	Nutraceuticals modulate inflammatory pathways in cancer	Multiple pathway modulation by dietary compounds; comprehensive mechanisms	Pecan constituents fit paradigm of multi-targeted nutraceutical cancer interventions
Ren et al., 2010 [54]	Dietary polyphenols, inflammation, and cancer connections	Polyphenols suppress inflammatory cytokines; cancer prevention mechanisms	Pecan polyphenols align with established anti-inflammatory cancer prevention strategies
Wang & DuBois, 2010 [55]	COX-2 role in intestinal inflammation and colorectal cancer	COX-2 critical in colorectal cancer; therapeutic target validation	Pecan COX-2 inhibitory activity relevant to colorectal cancer prevention
Howe et al., 2001 [56]	Cyclooxygenase-2 implications for cancer prevention and therapy	COX-2 promotes tumorigenesis; inhibition prevents cancer; therapeutic potential	Pecan tocopherols and phenolics targeting COX-2 could contribute to cancer prevention
Pandurangan et al., 2016 [57]	Dietary phytochemicals targeting cancer stem cells	Phytochemicals modulate Wnt, EMT pathways; cancer stem cell targeting	Pecan compounds may affect cancer stem cell populations through pathway modulation
Keshet & Seger, 2010 [58]	MAP kinase signaling cascades; hundreds of components	MAPK complexity; diverse physiological functions; cancer relevance	Pecan polyphenols likely modulate MAPK signaling; complex context-dependent effects
Yu et al., 2009 [59]	STAT3 in cancer inflammation and immunity	STAT3 promotes inflammation-driven cancer; therapeutic target	Pecan compounds may inhibit STAT3; anti-inflammatory anticancer mechanism
Zhang et al., 2012 [60]	Targeting STAT3 in cancer: mechanisms and drugs	STAT3 inhibition strategies; anticancer potential	Pecan polyphenols could contribute to STAT3 targeting; complementary approach
Kelley et al., 2019 [61]	NLRP3 inflammasome: activation and regulation mechanisms	Inflammasome in inflammatory diseases; IL-1β and IL-18 processing	Pecan polyphenols may inhibit inflammasome; emerging anti-inflammatory mechanism
Tlsty and Coussens, 2006 [62]	Tumor stroma and regulation of cancer development	Tumor microenvironment complexity; stromal-cancer cell interactions	Pecan effects likely extend beyond cancer cells; microenvironment modulation needed for full efficacy

**Table 3:** Inclusion and exclusion criteria.

Criteria	Inclusion	Exclusion
Publication Type	Peer-reviewed journal articles, theses, and conference proceedings with full text	Editorials, commentaries, book chapters, preprints without peer review
Language	English only	Non-English publications
Study Period	2015–2025	Studies published before 2015
Plant Part	Pecan seed/kernel or seed oil extracts	Studies on leaves, shells, bark, or unrelated tree species
Study Focus	Bioactive compounds, antioxidant activity, anti-inflammatory potential, anticancer mechanisms, or modulation of cell signaling	Studies unrelated to inflammation, oxidative stress, or cancer
Experimental Design	In vitro, in vivo, or analytical studies examining molecular or biochemical mechanisms	Reviews lacking original data or studies with no mechanistic evaluation
Endpoints	Antioxidant markers, cytokine modulation, phosphorylation pathways, gene regulation, apoptosis, or related cancer biomarkers	Studies assessing only nutritional composition without mechanistic evaluation
